# Supplementary material for: Encoding edge type information in graphlets
Source: PLoS One. 2022 Aug 26;17(8):e0273609. doi: 10.1371/journal.pone.0273609 (PMC9416998; doi:10.1371/journal.pone.0273609)
Supplement: Attachment — Submitted filename: Rebuttal_Letter_PlosOne22.pdf [file pone.0273609.s001.pdf]

PONE-D-22-15936
Encoding edge type information in graphlets
PLOS ONE

Rebuttal Letter

Dear Editor,

Thank you very much for handling our submission. We have now carefully revised the manuscript according to your and the reviewers' comments and suggestions.

The main changes include: (i) We have rephrased and revised all paragraphs that have overlapping texts with our previously published conference paper; (ii) We have restructured and extended the discussion about the current state of the art and related research and it is now a separate section in the article; (iii) We have included and discussed a radar chart in experiment part to better illustrate the findings; (iv) We have added a subsection discussing the limitations and future directions and included more references; (iv) We have added more discussions and examples throughout the article to better introduce and explain our methods. Enclosed please find point-to-point responses to the reviewing reports.

Kind regards,
Mingshan Jia, Maité Van Alboom, Liesbet Goubert, Piet Bracke, Bogdan Gabrys and Katarzyna Musial

---

Reviewer 1

Reviewer Comment C 1.1 — The brief methodology should be given in the abstract; the obtained results should be included in the abstract. The results should be included in terms of improvement ratio between the proposed and existing works.

Authors response: We have revised the abstract to include more discussion about the proposed approaches and findings. The experiment results include performance and time comparisons of multiple methods, which are presented and discussed thoroughly in Section 5.

Reviewer Comment C 1.2 — The structure of the manuscript need to be included at the end of the introduction section; a related work section should be included in the manuscript.

Authors response: In the revised manuscript, we have added a paragraph to illustrate the structure of the manuscript at the end of the introduction section. We have also restructured and extended the discussion of related works, and it is now a separate section in the article (Section 6).

Reviewer Comment C 1.3 — More justification is required in the section of Typed-Edge Graphlet Degree Vector.

Authors response: To better illustrate the idea of the proposed approach, we added more discussion about the existing problems in analysing typed-edge networks and discussed the proposed method in more depth (both in Section 1 and Section 3).

Reviewer Comment C 1.4 — For more readability, more visual examples are required in the section of Experiments and Analysis.

Authors response: We have included a radar chart in the experiment to better illustrate the findings from the traditional graphlet degree vector approach. To increase the readability of this section, we further revised the discussion about the findings from applying TyE-GDV in the analysis.

Reviewer Comment C 1.5 — The limitation of this work should be clearly included in the conclusion section. Also, future work need to be included in this section.

Authors response: In the revised manuscript, a subsection of Limitations and Future Directions is added at the end of Section 5.

Reviewer Comment C 1.6 — References need to be updated from literature (2022).

Authors response: In the revised manuscript, more references are now included when discussing the limitations and future directions, including three papers about directed graphlets [1–3], one paper on the notion of temporal graphlets [4], two papers about predicting future links with both network structure and attributes [5, 6], and three papers about the potential applications in network alignment [7] and in graph neural networks [8, 9].

Moreover, in related work discussion, two more references are added when introducing the approach that preserves the hierarchical property and edge attributes [10, 11], and in the experiment section, we add another reference that aims to analyse the perception of pain using network topological feature [12].

Reviewer 2

Reviewer Comment C 2.1 — While the code is made available on GitHub, the data is not (at least not obviously to be found in the GitHub repository). For means of reproducibility it should be provided.

Authors response: As this dataset concerns sensitive information of vulnerable people, it is not publicly available at this stage. This is also the policy of Ghent Health Psychology Lab. However, interested researchers and readers can contact author Maité Van Alboom (Maite.vanalboom@ugent.be) in order to obtain access to the data.

Reviewer Comment C 2.2 — Algorithm 3: The variables τ_{o_6}, τ_{o_9} etc. used in the algorithm are not defined, neither in the algorithm nor in the text.

Authors response: According to the comment, the variables have been updated and defined in Algorithm 3.

Reviewer Comment C 2.3 — Lines 135 and 137: It should read ”from 0 to $|\mathcal{T}_e| - 1$” and ”from 0 to $|\mathcal{O}| - 1$”, respectively, to match the examples given within the same sentences.

Authors response: Indeed, according to the comment, it has been changed to from 0 to $|\mathcal{T}_e| - 1$, and from 0 to $|\mathcal{O}| - 1$.

Reviewer Comment C 2.4 — Line 160f: It would be helpful to add a reference to Fig. 2, which shows the 7 orbits of TyE-EGDV.

Authors response: In the revised manuscript, a reference to Fig. 2 has been added to illustrate the point.

Reviewer Comment C 2.5 — Equations (1) and (2): The derivation of the formulae should be briefly explained, in particular since (1) is claimed to be a correction of a result previously published in [23].

Authors response: In the revised manuscript, more discussion about the derivation of the formulae has been added. We also included a simple example to present the problem in the original formula.

Reviewer Comment C 2.6 — Fig. 4: The figure could be drawn as a radar chart, which might be more familiar to the usual reader than a parallel coordinates plot. The difference between the pain grades with respect to orbit types might also be more obvious in a radar chart. For Fig. 5 parallel coordinate plots are probably more appropriate due to the large number of attributes (edge types).

Authors response: As recommended, the parallel coordinates plot has been changed to a radar chart, which would be more familiar to the general public (Fig 1).

Reviewer Comment C 2.7 — References: Starting from ref. [17], page numbers are missing for most references. Also the formatting of reference entries varies, e.g., regarding the capitalization of journal titles. The whole reference section should be thoroughly proofread and amended where necessary.

Authors response: In the revised manuscript, we have added the missing page numbers for all references and amended the formatting with the first letter capitalised for journal and conference titles.

Academic Editor

Editor Comment 1 — Please ensure that your manuscript meets PLOS ONE’s style requirements, including those for file naming.

Authors response: We have carefully checked our manuscript to make sure that it meets PLOS ONE’s style requirements.

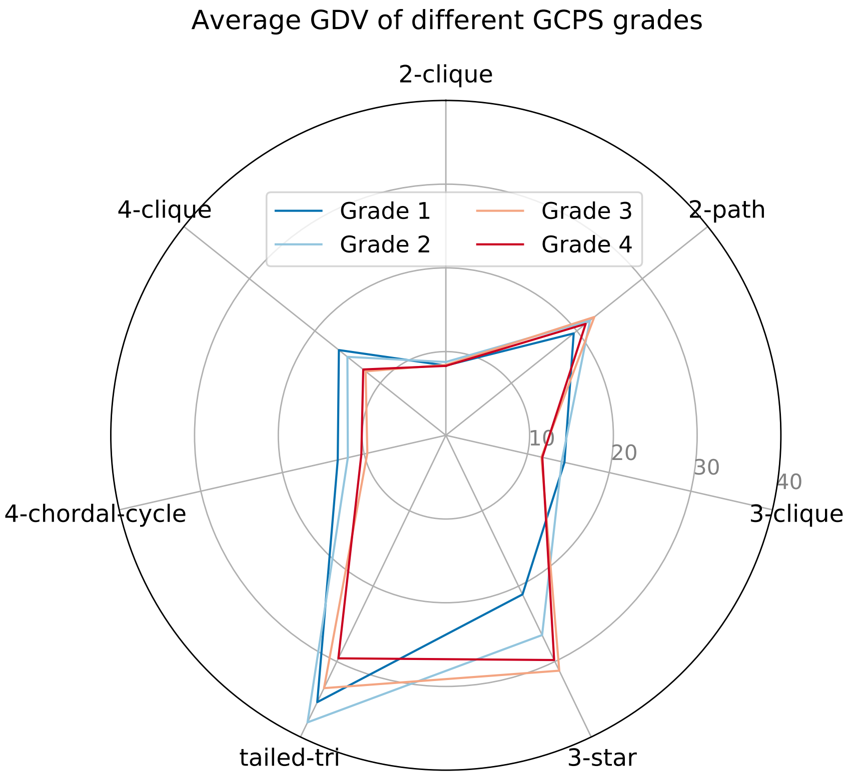

Figure 1: Radar chart of average GDV of different GCPS grades. Each spoke represents the average number of graphlets belonging to that type.

Editor Comment 2 — We noticed you have some minor occurrence of overlapping text with the following previous publication(s), which needs to be addressed: `https://link.springer.com/chapter/10.1007/978-3-030-93409-5_43`

In your revision ensure you cite all your sources (including your own works), and quote or rephrase any duplicated text outside the methods section.

Authors response: In the revised manuscript, we have thoroughly rephrased and revised all contents that have overlapping texts with our previously published conference paper.

References

[1] A. Sarajlić, N. Malod-Dognin, Ö. N. Yaveroğlu, and N. Pržulj, "Graphlet-based characterization of directed networks," *Scientific reports*, vol. 6, no. 1, pp. 1–14, 2016.

[2] D. Aparicio, P. Ribeiro, and F. Silva, "Extending the applicability of graphlets to directed networks," *IEEE/ACM Transactions on Computational Biology and Bioinformatics*, vol. 14, no. 6, pp. 1302–1315, 2016.

[3] I. Trpevski, T. Dimitrova, T. Boshkovski, N. Stikov, and L. Kocarev, "Graphlet characteristics in directed networks," *Scientific Reports*, vol. 6, no. 1, pp. 1–8, 2016.

[4] Y. Hulovatyy, H. Chen, and T. Milenković, "Exploring the structure and function of temporal networks with dynamic graphlets," *Bioinformatics*, vol. 31, no. 12, pp. i171–i180, 2015.

[5] Z. Yin, M. Gupta, T. Weninger, and J. Han, "Linkrec: a unified framework for link recommendation with user attributes and graph structure," in *Proceedings of the 19th international conference on World wide web*, 2010, pp. 1211–1212.

[6] N. Z. Gong, A. Talwalkar, L. Mackey, L. Huang, E. C. R. Shin, E. Stefanov, D. Song *et al.*, "Jointly predicting links and inferring attributes using a social-attribute network (san)," *arXiv preprint arXiv:1112.3265*, 2011.

[7] L. Ma, Z. Shao, L. Li, J. Huang, S. Wang, Q. Lin, J. Li, M. Gong, and A. K. Nandi, "Heuristics and metaheuristics for biological network alignment: A review," *Neurocomputing*, 2022.

[8] G. Bouritsas, F. Frasca, S. P. Zafeiriou, and M. Bronstein, "Improving graph neural network expressivity via subgraph isomorphism counting," *IEEE Transactions on Pattern Analysis and Machine Intelligence*, 2022.

[9] P. Barceló, F. Geerts, J. Reutter, and M. Ryschkov, "Graph neural networks with local graph parameters," *Advances in Neural Information Processing Systems*, vol. 34, pp. 25 280–25 293, 2021.

[10] M. Nickel and D. Kiela, "Poincaré embeddings for learning hierarchical representations," *Advances in neural information processing systems*, vol. 30, 2017.

[11] M. Chen and C. Quirk, "Embedding edge-attributed relational hierarchies," in *Proceedings of the 42nd International ACM SIGIR Conference on Research and Development in Information Retrieval*, 2019, pp. 873–876.

[12] M. Jia, M. Van Alboom, L. Goubert, P. Bracke, B. Gabrys, and K. Musial, "Analysing egocentric networks via local structure and centrality measures: A study on chronic pain patients," in *2022 International Conference on Information Networking (ICOIN)*. IEEE, 2022, pp. 152–157.
