# Supplementary material for: Encoding edge type information in graphlets
Source: PLoS One. 2022 Aug 26;17(8):e0273609. doi: 10.1371/journal.pone.0273609 (PMC9416998; doi:10.1371/journal.pone.0273609)
Supplement: Attachment — Submitted filename: Rebuttal_letter_R2.pdf [file pone.0273609.s002.pdf]

PONE-D-22-15936R1
Encoding edge type information in graphlets
PLOS ONE

Rebuttal Letter

Dear Editor,

Thank you again for handling our submission. We have carefully revised the manuscript according to your and the reviewers' comments and suggestions.

The main changes include: (i) We have repositioned the section of Related Work, and now it follows the Introduction Section; (ii) We have carefully checked our reference list to ensure that it is complete and correct. Enclosed please find point-to-point responses to the reviewing reports.

Kind regards,
Mingshan Jia, Maité Van Alboom, Liesbet Goubert, Piet Bracke, Bogdan Gabrys and Katarzyna Musial

---

Reviewer 1

Reviewer Comment C 1.1 — The section of related work needs to be moved after the Introduction Section.

Authors response: As suggested, we have revised the manuscript, and now the Related Work Section follows the Introduction Section.

Academic Editor

Editor Comment 1 — Although the quality of this work has been improved a lot after revision, one reviewer still has some minor concerns about the paper, please address them for publication.

Authors response: Reviewer #1 suggested us to move the Related Work Section after the Introduction Section. We have adopted the suggestion and made the changes accordingly.
